# Clinical Study on the Combined Therapy of Sinew Acupuncture and Low‐Frequency Repetitive Transcranial Magnetic Stimulation for Hemiplegic Shoulder Pain

**DOI:** 10.1002/brb3.70913

**Published:** 2025-12-22

**Authors:** Lintong Zhu, Hongwei Zhai, Caixia Su, Lei Qin, Zhaoxue Li, Zunke Gong, Mi Wang

**Affiliations:** ^1^ Department of Physiotherapy Xuzhou Central Hospital Xuzhou China; ^2^ Department of Spinal Cord Injury Rehabilitation Xuzhou Rehabilitation Hospital Xuzhou China; ^3^ Department of Rehabilitation Xuzhou Central Hospital Xuzhou China; ^4^ Department of Neurological Rehabilitation Xuzhou Rehabilitation Hospital Xuzhou China

**Keywords:** acupuncture, combined therapy, hemiplegia, shoulder pain, stroke, transcranial magnetic stimulation

## Abstract

**Objective:**

To observe the clinical efficacy of combining sinew acupuncture with low‐frequency repetitive transcranial magnetic stimulation (rTMS) for treating hemiplegic shoulder pain.

**Methods:**

A total of 90 patients with hemiplegic shoulder pain were randomly divided into three groups: the sinew acupuncture group, the rTMS group, and the combined group (30 patients each). All received conventional symptomatic medication and routine interventions. The sinew acupuncture group received subcutaneous shallow needling on the affected side; the rTMS group received 1 Hz rTMS stimulation on the contralateral M1 area of the cerebral cortex; and the combined group underwent both treatments. Pain severity, upper limb mobility, and activities of daily living were assessed using the Visual Analogue Scale (VAS), the Fugl‐Meyer Assessment (FMA) of Upper Extremity, and the Modified Barthel Index (MBI) before and after 4 weeks of treatment.

**Results:**

After treatment, all groups demonstrated substantial improvements in VAS, FMA, and MBI scores compared with baseline (*p* < 0.05). The combined therapy group exhibited superior improvements in pain reduction, upper limb function, and activities of daily living compared with the other two groups (*p* < 0.05).

**Conclusion:**

Both sinew acupuncture and 1 Hz rTMS are effective in treating hemiplegic shoulder pain when used independently. Their combined application substantially reduces pain and enhances shoulder function and activities of daily living in patients, representing a valuable clinical approach.

## Graphical abstract

This study investigated the effect of sinew acupuncture combined with low‐frequency repetitive transcranial magnetic stimulation (rTMS) in the treatment of hemiplegic shoulder pain. Sinew acupuncture relieves local myofascial tension and pain through superficial puncture of the subcutaneous fascial layer; rTMS regulates the imbalance between the hemispheres after stroke and promotes neuroplasticity by inhibiting the overexcitation of the motor cortex (M1 area) on the unaffected side of the brain. The synergistic effect significantly improved shoulder pain (decreased VAS score), upper limb motor function (increased FMA score), and ability to perform activities of daily living (increased MBI score). The combination therapy has shown superior efficacy over monotherapy in the short term and has good clinical translational prospects.

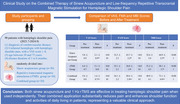

## 1 Introduction

Stroke remains the second leading cause of death and disability globally, affecting over 15 million people each year (Ballester et al. 2021). Approximately three‐quarters of stroke patients experience impaired upper limb function, which substantially diminishes quality of life. Shoulder pain, a common poststroke complication, not only exacerbates patient suffering but also severely hinders rehabilitation progress (Morris et al. 2013). Many patients recover slowly, with persistent shoulder pain often resulting in secondary disabilities (Morris et al. 2013).

The shoulder joint, a complex system comprising bony structures, ligaments, and muscle tissues, functions under normal biomechanical conditions. However, following hemiplegia, the elasticity and tension balance of the shoulder myofascia become disrupted, compromising joint stability. Localized myofascial dysfunction contributes to shoulder impairment, soft tissue injury, and pain. Studies have identified pathological changes associated with hemiplegic shoulder pain, such as shoulder impingement syndrome, long head of biceps tendon injury, and rotator cuff tears. These conditions are closely linked to pain and impaired motor function in affected patients (Yi 2021).

Sinew acupuncture, a novel and painless needling technique, involves inserting needles into the fascial layer to generate a piezoelectric effect and, consequently, bioelectricity. When directed to injured tissues, this bioelectricity produces a counter‐piezoelectric effect, reducing fascial tension, dilating blood vessels, enhancing metabolism, and raising the local pain threshold to alleviate pain (Xi et al. 2021).

In parallel, numerous studies have shown that repetitive transcranial magnetic stimulation (rTMS), a noninvasive brain stimulation method, uses coils placed near the scalp to produce a magnetic field of specific frequency, thereby modulating neuronal activity. It can reduce inflammation, inhibit neuronal apoptosis, repair damaged blood–brain barriers, and enhance neural plasticity by regulating synapse‐associated proteins and genes while also strengthening excitatory synaptic transmission. Furthermore, rTMS promotes neuronal migration and reduces neuronal loss by increasing the expression of matrix‐derived factors and chemokine receptors in the peri‐infarct cortex, ultimately alleviating pain and improving limb function. Low‐frequency (1 Hz) rTMS has been found to be relatively safe and more effective (Lefaucheur et al. 2020).

This study introduces a novel combined therapeutic approach that integrates sinew acupuncture with low‐frequency rTMS for treating hemiplegic shoulder pain. Sinew acupuncture, a minimally invasive technique, targets the fascial layer to improve local biomechanics and reduce pain through the generation of a piezoelectric effect, which enhances local metabolism and increases the pain threshold (Xi et al. 2021). In contrast, rTMS modulates cortical excitability, facilitates neuroplasticity, and supports neural repair processes (Lefaucheur et al. 2020). Low‐frequency (1 Hz) rTMS has shown particular efficacy in reducing inflammation, preventing neuronal apoptosis, and improving motor function (Lefaucheur et al. 2020).

Despite the high prevalence and substantial impact of hemiplegic shoulder pain, current treatment options are limited and often produce suboptimal outcomes. Traditional interventions such as pharmacotherapy, physical therapy, and nerve blocks are commonly used but are frequently associated with adverse effects, limited efficacy, or procedural risks (T. Zhang et al. 2023; Yue et al. 2022). Consequently, there is a pressing need to investigate novel and more effective therapeutic strategies for this debilitating condition.

## 2 Participants and Methods

### 2.1 Participants and Grouping

This study was designed and reported in accordance with the consolidated standards of reporting trials guidelines to ensure transparency and completeness. It was conducted as a prospective, randomized clinical trial. Patients were assigned randomly to one of three groups (sinew acupuncture group, rTMS group, and combined group) using a random number table method. Efficacy assessments were conducted by rehabilitation therapists who were blinded to group assignments to ensure objective outcome evaluation. This parallel design enabled independent follow‐up of each group and robust comparisons between the different interventions at the end of the treatment period.

A total of 90 patients with hemiplegic shoulder pain admitted to the Department of Rehabilitation at Xuzhou Rehabilitation Hospital between July 2023 and September 2024 who met the inclusion criteria were enrolled. Using the random number table method, patients were randomly allocated to the acupuncture group, the rTMS group, or the combined group, with 30 patients in each.

The inclusion criteria were as follows: (1) diagnosis of cerebrovascular disease according to the *Diagnostic Criteria of Cerebrovascular Diseases in China (Version 2019)* (Chinese Society of Neurology 2019); (2) hemiplegia affecting one side; (3) shoulder pain on the hemiplegic side, with a Visual Analogue Scale (VAS) score ≥ 4; (4) age between 30 and 75 years; (5) disease duration of 1–6 months; (6) clear consciousness, stable condition, and ability to communicate; (7) signed informed consent provided by the patient or their legal guardian.

The exclusion criteria were as follows: (1) fixed contracture of the shoulder joint or presence of local metal implants; (2) severe comorbidities involving the heart, liver, kidneys, endocrine or hematopoietic systems; (3) substantial musculoskeletal injuries or other conditions affecting upper limb mobility; (4) history of seizures or atrial fibrillation; (5) coagulation disorders or local skin infections; (6) participation in other clinical trials that could influence study outcomes; (7) noncompliance, withdrawal or adverse events preventing continued treatment; (8) intolerance of or objection to acupuncture; (9) contraindications to rTMS, such as cardiac pacemakers, intracranial metal implants or other internal metal devices; (10) previous rTMS treatment; (11) pregnancy or breastfeeding; (12) use of systemic analgesics (e.g. opioids or NSAIDs) within 48 h prior to study initiation; (13) first stroke only, to avoid confounding from recurrent stroke events; (14) severe psychiatric disorders that could affect participation or data interpretation; (15) preexisting neurological disorders other than stroke (e.g., peripheral neuropathy, multiple sclerosis) that could influence pain perception or motor function.

The general characteristics of the three groups showed no statistically significant differences (*p* > 0.05), indicating baseline comparability. The study was approved by the Ethics Committee of Xuzhou Rehabilitation Hospital (XK‐LW‐20230529‐010).

### 2.2 Treatment Methods

All groups received routine interventions, including local hot compresses, transcutaneous electrical nerve stimulation, stretching training, and symptomatic management of blood pressure, blood glucose, and lipid levels, as well as neural nutrition. In addition to routine treatments, the sinew acupuncture group received local sinew acupuncture for hemiplegic shoulder pain, the rTMS group received 1 Hz rTMS treatment on the healthy cerebral hemisphere, and the combined group received local sinew acupuncture followed by 1 Hz rTMS treatment on the healthy cerebral hemisphere. Specific therapies were as follows:

#### 2.2.1 Sinew Acupuncture Treatment

Sinew acupuncture (Cai et al. 2023) is a minimally invasive, superficial needling technique that targets the fascial layer of the body. Unlike traditional acupuncture, which involves deeper needling and often elicits a “De‐qi” sensation characterized by sourness and distension, sinew acupuncture focuses on superficial insertion. This technique is designed to be painless and is particularly effective in treating conditions associated with fascial tension and myofascial pain.

The patient was positioned either in a seated posture or on their unaffected side. Muscular knots and tender points near the shoulder region on the hemiplegic side were palpated, and insertion points were determined based on these locations along the meridians. Following routine disinfection, a 0.30 × 40 mm cannula needle was inserted at the meridian points, penetrating 30–35 mm subcutaneously towards the muscular knots and tender points. A light sensation without obstruction was essential during insertion. Patients were then instructed to actively move the shoulder or perform assisted passive movements, with the needle retained for 20 min. Treatment was administered once per day, 5 days a week, for 4 weeks.

#### 2.2.2 rTMS Treatment

Prior to initiating rTMS treatment (Wang et al. 2017), the resting motor threshold (RMT) was determined for each participant. The RMT was defined as the minimum stimulation intensity required to elicit a motor‐evoked potential (MEP) of at least 50 µV in the target muscle in at least 5 out of 10 trials. Participants were seated in a comfortable, relaxed position, and the target muscle for MEP recording was either the abductor pollicis brevis or the first dorsal interosseous on the side contralateral to the stimulated hemisphere.

Single‐pulse TMS was delivered using a Magstim RAPID2 TMS system (Magstim, UK) with a figure‐of‐eight coil (diameter approximately 7 cm; maximum output intensity 2.0 T). The stimulation intensity gradually increased from a low baseline, with a single pulse delivered every 5 s. MEPs were confirmed either visually, by observing thumb twitching, or via electromyography (EMG) to record muscle activity. The RMT was established as the lowest intensity at which MEPs of at least 50 µV were elicited in 5 out of 10 trials.

Based on the RMT, the rTMS treatment parameters were as follows: frequency of 1 Hz (low‐frequency rTMS), intensity at 90% of RMT, and a total of 900 pulses per session. Pulses were delivered in trains of 30 pulses, with 30‐s intervals between trains, resulting in a total session duration of approximately 15 min. Treatment was administered once per day, 5 days a week, for 4 weeks.

During each session, participants remained seated in a relaxed position. The coil was positioned over the contralateral primary motor cortex (M1), guided by the international 10/20 EEG system. rTMS was delivered in 30‐pulse trains at 1 Hz, with 30‐s intervals, totaling 900 pulses per session. This protocol ensured that the rTMS treatment was both scientifically rigorous and clinically appropriate.

EMG was used during rTMS treatment to record MEPs for the precise determination of the RMT at baseline and was recalculated weekly to account for variability. The stimulation target was the contralateral M1 corresponding to the hand area, as identified using the international 10/20 EEG system. To ensure safety, participants were screened for contraindications, closely monitored during sessions, provided with earplugs for hearing protection, and followed up regularly to assess any delayed adverse effects.

### 2.3 Efficacy Evaluation Methods

Demographic and baseline clinical characteristics of the participants were collected at the beginning of the study. These data included age, gender, type of stroke (ischemic or hemorrhagic), time since stroke onset, and affected side. Before treatment and after 4 weeks, VAS scores, upper limb FMA scores, and MBI scores were recorded by rehabilitation therapists who were unaware of patient groupings. The VAS reflects pain intensity, whereas the Fugl‐Meyer Assessment (FMA) evaluates upper limb motor function.

#### 2.3.1 Visual Analogue Scale

The VAS (Andres et al. 2024) ranges from 0 to 10, with 0 indicating no pain and 10 indicating severe pain. Patients were instructed to mark the scale according to their subjective perception of pain. Additionally, the upper limb function was assessed using the simplified VAS of Upper Extremity (X. X. Zhang et al. 2024), which comprises 10 items and 33 sub‐items, each scored from 0 to 2 points, with a total maximum score of 66 points. Higher scores on this assessment indicate stronger motor abilities of the limbs.

#### 2.3.2 Modified Barthel Index

The MBI (Pournajaf et al. 2023) includes 10 indicators, each divided into five levels of severity, covering activities such as eating, ambulation, grooming, dressing, bowel and bladder control, toileting, bathing, stair climbing, and bed‐to‐chair transfer. The scoring system allows a maximum of 100 points, with higher scores reflecting greater independence in daily life activities.

### 2.4 Statistical Methods

All data were analyzed using SPSS version 25.0 (SPSS Inc., Chicago, IL, USA). Before conducting the main analyses, assumptions of normality and homogeneity of variance were assessed. Normality was evaluated using the Shapiro–Wilk test, and homogeneity of variance was assessed using Levene's test.

For within‐group comparisons, paired *t*‐tests were used when the data met the assumption of normality; otherwise, the Wilcoxon signed‐rank test was applied. Between‐group comparisons were conducted using one‐way ANOVA when assumptions were met; otherwise, the Kruskal–Wallis *H* test was used, followed by post hoc pairwise comparisons with Bonferroni correction to adjust for multiple comparisons.

Effect sizes were calculated and reported alongside p‐values to reflect the magnitude of the observed effects. For *t*‐tests, Cohen's *d* was reported, and for ANOVA, eta‐squared (*η* ^2^) was used to indicate the proportion of variance explained by the independent variable.

The sample size for this study was determined based on a power calculation aiming for 80% power (*β* = 0.20) to detect a medium effect size (Cohen's *d* = 0.5) at a significance level of *α* = 0.05. This calculation was based on previous studies in the field, which reported similar effect sizes for interventions targeting hemiplegic shoulder pain. We aimed to enroll 30 participants per group to ensure sufficient statistical power while considering the feasibility and resources available for the study.

## 3 Results

### 3.1 Baseline Characteristics of Participants

Table 1 presents the baseline demographic and clinical characteristics of participants across the three treatment groups. The groups were well‐balanced in terms of age, gender, disease duration, stroke type, and hemiplegia type. No significant differences were observed among the groups for any of these variables (*p* > 0.05), indicating that the randomization process was successful and the groups were comparable at baseline.

**TABLE 1:** Comparison of general data among the three groups (*n* = 90).

Characteristic	Sinew acupuncture group (*n* = 30)	rTMS Group (*n* = 30)	Combined group (*n* = 30)	*F*/*χ* ^2^	*p* value
Number of cases	30	30	30	—	—
Gender (male/female)	14/16	13/17	18/12	1.203	0.305
Age (years)	61.9 ± 6.5	60.97 ± 7.0	59.3 ± 5.7	0.062	0.940
Disease course (days)	56.9 ± 8.7	57.5 ± 8.4	57.7 ± 9.6	2.494	0.287
Stroke type (hemorrhagic/ischemic)	12/18	15/15	17/13	1.867	0.393
Hemiplegia type (left/right)	13/17	14/16	17/13	1.156	0.561

### 3.2 Comparison of VAS, FMA, and MBI Scores Before and After Treatment

Table 2 shows the comparison of VAS, FMA, and MBI scores before and after treatment across the three groups. All groups demonstrated significant improvements in VAS, FMA, and MBI scores following treatment compared with pretreatment values (*p* < 0.001). The combined group exhibited the most pronounced improvements in all three outcome measures compared with the sinew acupuncture and rTMS groups (*p* < 0.05).

**TABLE 2:** Comparison of VAS, FMA, and MBI scores before and after treatment (score, *x* ± *s*).

	VAS score			FAM score			MBI score		
Group	Pretreatment	Posttreatment	*t*/*P*	Pretreatment	Posttreatment	*t*/*P*	Pretreatment	Posttreatment	*t*/*P*
Sinew acupuncture group	5.8 ± 0.9	2.1 ± 0.9^a^, ^b^	36.797/< 0.001	33.3 ± 2.3	43.7 ± 2.2^a^, ^b^	31.775/< 0.001	52.4 ± 3.8	64.9 ± 2.5^a^, ^b^	16.655/< 0.001
rTMS group	6.4 ± 0.7	5.1 ± 0.6^a^	52.150/< 0.001	33.1 ± 2.1	36.0 ± 1.9^a^	11.783/< 0.001	51.2 ± 4.5	58.1 ± 3.7^a^	15.140/< 0.001
Combined group	5.6 ± 0.8	1.6 ± 0.7^a^, ^b^, ^c^	39.072/<0.001	34.1 ± 2.2	49.4 ± 2.8^a^, ^b^, ^c^	25.918/< 0.001	49.9 ± 4.1	71.1 ± 4.8^a^, ^b^, ^c^	17.977/< 0.001
*F*	—	—	8.235	—	—	1.729	—	—	2.645
*p*	—	—	0.067	—	—	0.183	—	—	0.077

^a^ *p *< 0.05 when compared with pretreatment values within the same group.

^b^ *p *< 0.05 when compared with the rTMS group posttreatment.

^c^ *p *< 0.05 when compared with the sinew acupuncture group posttreatment.

VAS score: The sinew acupuncture group showed a significant reduction in VAS score from 5.8 ± 0.9 to 2.1 ± 0.9 (*t* = 36.797, *p* < 0.001). The rTMS group exhibited a reduction from 6.4 ± 0.7 to 5.1 ± 0.6 (*t* = 52.150, *p* < 0.000). The combined group had the most significant reduction, from 5.6 ± 0.8 to 1.6 ± 0.7 (*t* = 39.072, *p* < 0.001). The combined group's posttreatment VAS score was significantly lower than those of both the sinew acupuncture and rTMS groups (*p* < 0.05).

FMA score: The sinew acupuncture group's FMA score increased from 33.3 ± 2.3 to 43.7 ± 2.2 (*t* = 31.775, *p* < 0.001). The rTMS group's FMA score improved from 33.1 ± 2.1 to 36.0 ± 1.9 (*t* = 11.783, *p* < 0.000). The combined group's FMA score increased from 34.1 ± 2.2 to 49.4 ± 2.8 (*t* = 25.918, *p* < 0.001). The posttreatment FMA score of the combined group was significantly higher than that of both the sinew acupuncture and rTMS groups (*p* < 0.05).

Modified Barthel Index score: The sinew acupuncture group's MBI score rose from 52.4 ± 3.8 to 64.9 ± 2.5 (*t* = 16.655, *p* < 0.001). The rTMS group's MBI score increased from 51.2 ± 4.5 to 58.1 ± 3.7 (*t* = 15.140, *p* < 0.001). The combined group's MBI score improved from 49.9 ± 4.1 to 71.1 ± 4.8 (*t* = 17.977, *p* < 0.001). The posttreatment MBI score of the combined group was significantly higher than that of both the sinew acupuncture and rTMS groups (*p* < 0.05).

## 4 Discussion

This study found that posttreatment VAS, upper limb FMA, and MBI scores improved substantially in all three groups compared with pretreatment scores. Notably, the combined group exhibited substantially greater improvements in these scores than both the sinew acupuncture group and the rTMS group, suggesting that sinew acupuncture combined with rTMS is more effective in mitigating shoulder pain in hemiplegic patients, enhancing shoulder mobility, and improving activities of daily living.

Traditional Chinese acupuncture has been proven effective in treating hemiplegic shoulder pain; however, the practice typically requires eliciting a “De‐qi (Acuesthesia)” sensation, characterized by sourness and distension. It also involves prolonged needle retention, which may induce fear in patients during treatment, along with adverse reactions such as pain, localized bruising, bent needles, needle stagnation, and fainting. In contrast, sinew acupuncture is an innovative superficial needling technique that, compared with floating needling, offers a minimally painful, safe, simple, cost‐effective, and efficient approach.

When myofascial tension is excessively high or reflexive contractions occur, the lowered pain threshold of free nerve endings leads to disorganized collagen fibers, resulting in local and referred pain even with minimal compression. When these underlying nerve endings, lymphatic vessels, and blood vessels in the fascial layer are in a “contracted” state, inflammation and swelling cause increased local pressure, stimulating receptors and producing pain (T. Zhang et al. 2023; Yue et al. 2022; Gibbons 2021; Song et al. 2022).

Sinew acupuncture may facilitate lymphatic drainage through micro‐lifting of the skin; this deformation increases interstitial spaces, alleviates inflammation at the injury site, and reduces fascial tension. When combined with physiological movement, it supports the realignment of collagen fibers along lines of tension (Guan 2018). Additionally, clinical studies (Wu 2023) have reported that sinew acupuncture substantially reduces pain scale scores and provides immediate analgesic effects.

Repetitive rTMS is an emerging neuro‐intervention technique for the brain, utilizing transient magnetic fields generated by coils placed on the scalp, which produce immediate electrical pulses that directly stimulate the local brain region beneath the coil. The magnetic pulses induced by the coil generate an electric field in the cortex, leading to neuronal activation during depolarization of pyramidal cells and inhibitory interneurons. Retrospective analyses by international researchers (Ojha et al. 2023) suggest that rTMS operates based on an interhemispheric competition model, modulating cortical excitability poststroke via the transcallosal pathway to inhibit the contralateral motor cortex, thereby enhancing the plasticity of damaged neural components.

The stroke‐induced imbalance between cerebral hemispheres results in excessive inhibition of the impaired hemisphere by the contralateral one. High‐frequency rTMS (≥ 5 Hz) has been shown to increase neuronal excitability within the M1, whereas low‐frequency stimulation (< 5 Hz) can suppress such excitability (Safdar et al. 2023). Li et al. (2023) demonstrated that combining 10 Hz rTMS with electroacupuncture targeting the cortical M1 region produces superior analgesic outcomes when high‐frequency rTMS is used. Another clinical trial indicated that 1 Hz rTMS may improve spasticity in the upper limb of hemiplegic patients by reinforcing resting‐state functional connectivity between the sensorimotor areas of both hemispheres, potentially facilitating neurogenesis and neuroplasticity essential for the rehabilitation of disrupted neural networks (Chen et al. 2024).

Additionally, studies incorporating surface EMG and three‐dimensional gait analysis (Huang et al. 2024) have shown that 1 Hz rTMS stimulation of the M1 region modifies the electromyographic activity of the tibialis anterior and rectus femoris, increases step length, speed, and frequency, and thereby improves lower extremity motor and balance functions in stroke patients (Huang et al. 2024).

The minimal clinically important difference (MCID) for pain reduction using the VAS is commonly considered to be a reduction of at least 2 points (Farrar et al. 2001). In our study, the combined group showed a mean reduction in VAS score from 5.6 ± 0.8 to 1.6 ± 0.7, representing a mean reduction of 4.0 points. This exceeds the MCID, indicating that the pain reduction experienced by participants in the combined group is not only statistically significant but also clinically meaningful.

The MCID for the FMA score, which measures upper limb motor function, is reported to be approximately 5 points (Hijikata et al. 2020). The combined group exhibited a mean increase in FMA score from 34.1 ± 2.2 to 49.4 ± 2.8, representing a mean improvement of 15.3 points. This improvement is well above the MCID, suggesting that the observed enhancement in upper limb function is clinically significant.

The MCID for the MBI score, which assesses activities of daily living, is approximately 5 points (Linacre et al. 1994). The combined group showed a mean increase in MBI score from 49.9 ± 4.1 to 71.1 ± 4.8, a mean improvement of 21.2 points. This improvement far exceeds the MCID, indicating that the combined therapy led to substantial and clinically meaningful improvements in participants’ daily living activities.

The combined therapy of sinew acupuncture and low‐frequency rTMS not only demonstrated superior efficacy in reducing pain and improving upper limb function and activities of daily living compared with either treatment alone but also achieved improvements exceeding the MCID for each outcome measure. This suggests that the observed effects are not only statistically significant but also clinically relevant, supporting the potential clinical application of this combined therapy for patients with hemiplegic shoulder pain.

The therapeutic mechanisms of combined sinew acupuncture and rTMS can be interpreted through both traditional and modern biomedical frameworks. From the perspective of Traditional Chinese Medicine (TCM), sinew acupuncture operates on the principle of regulating “defensive qi” (wei qi) within the meridian tendon system (jing jin)—a concept describing the body's superficial protective network. Classical TCM texts attribute its immediate analgesic effects to restoring the “swift and smooth” movement of qi along these fascial pathways (Xi et al. 2021; Cai et al. 2023). Modern neurophysiological studies complement this view by demonstrating that superficial fascial needling activates type I afferent fibers (muscle spindles and tendon organs), which modulate spinal cord nociceptive processing through segmental inhibition of wide dynamic range neurons (Liu et al. 2024). This biomechanical intervention synergizes with 1 Hz rTMS's established neuromodulatory effects: the low‐frequency stimulation reduces pathological cortical hyperactivity in the contralesional M1 area through transcallosal inhibition (Cao 2023; Mahmoud et al. 2023), while simultaneously enhancing functional connectivity within the ipsilesional sensorimotor network (Chen et al. 2024). The observed clinical superiority of the combined therapy likely stems from this dual‐pathway approach, where sinew acupuncture addresses peripheral fascial dysfunction (evidenced by piezoelectric effects on local microcirculation; Guan 2018; Tang et al. 2024), while rTMS targets central sensitization mechanisms (supported by fMRI studies showing normalized thalamocortical rhythms; Lefaucheur et al. 2020). Importantly, while TCM theory provides a holistic explanatory model for clinical observations, the neurophysiological mechanisms offer testable hypotheses for future research. This integrative perspective underscores the importance of evaluating traditional interventions through both their historical theoretical frameworks and contemporary scientific paradigms.

The unique aspect of our study lies in the synergistic combination of these two therapies. By integrating peripheral fascial manipulation with central cortical stimulation, we aim to address both the local biomechanical and central neurophysiological components of hemiplegic shoulder pain. This combined approach is expected to offer a more comprehensive and effective treatment strategy, potentially resulting in superior clinical outcomes compared with either therapy alone.

First, the small sample size may restrict the generalizability of the findings, highlighting the need for future studies with larger cohorts to improve external validity. Second, the evaluation of treatment efficacy was limited to a 4‐week period, which precludes conclusions regarding long‐term outcomes and recurrence rates. This limitation underscores the importance of conducting extended longitudinal follow‐up studies.

Third, the study design included randomization and evaluator blinding to reduce bias. However, the absence of a sham or placebo control group limits the ability to conclusively rule out placebo effects. Future trials should consider incorporating sham or placebo interventions to better control these effects. Additionally, although our randomization procedure used a random number table with allocation concealment, future studies should implement stratified block randomization (e.g., by stroke severity using NIHSS scores or baseline pain levels) to ensure balanced group characteristics, particularly given the heterogeneity of poststroke populations. Computerized allocation systems with concealed sequencing (such as REDCap's randomization module) would further minimize selection bias.

Although the 4‐week intervention period in our study was intended to assess the initial efficacy and safety of the combined therapy of sinew acupuncture and low‐frequency rTMS for hemiplegic shoulder pain, it is important to note that the durability of these effects beyond the intervention period remains unknown. Future research should incorporate longer‐term follow‐up assessments to evaluate the persistence of treatment effects. Follow‐up evaluations at 3‐, 6‐, and 12‐month post‐intervention would provide valuable insights into the long‐term benefits and potential recurrence of symptoms.

Furthermore, future studies could explore the potential of maintenance treatments or booster sessions to prolong the therapeutic effects observed during the initial intervention period. This would aid in determining the optimal treatment regimen for maximizing long‐term outcomes. Although the 4‐week period was appropriate for evaluating initial efficacy, extended follow‐up is essential for a comprehensive understanding of the long‐term benefits of combined therapy in patients with hemiplegic shoulder pain.

## 5 Conclusions

In conclusion, the combined therapy of sinew acupuncture and rTMS in patients with hemiplegic shoulder pain demonstrates enhanced effects, substantially alleviating shoulder pain and improving shoulder function and activities of daily living. This combined approach holds promise for clinical application and practice. Nevertheless, the study has certain limitations, including a small sample size, the absence of long‐term follow‐up on analgesic efficacy, and the lack of differentiation among underlying causes of shoulder pain. Future studies should consider incorporating MEP status as a biomarker of cortical motor function, utilizing functional imaging to visualize cortical excitability as an efficacy assessment criterion, and continuing to refine and optimize the research progressively.

## Author Contributions

L.Z. and H.Z. conceived of the study. C.S., L.Q., and Z.L. participated in its design and collected the data. Z.G. and M.W. participate in the data analysis and statistics. All authors helped to draft the manuscript. And all authors read and approved the final manuscript.

## Ethics Statement

This study was conducted in accordance with the Declaration of Helsinki and approved by the ethics committee of Xuzhou Rehabilitation Hospital (No. XK‐LW‐20230529‐010). All participants provided written informed consent before enrollment.

## Conflicts of Interest

The authors declare no conflicts of interest.

## Data Availability

All data generated or analyzed during this study are included in this published article.
